# Diagnosis and Management of Traumatic Subarachnoid Hemorrhage: Protocol for a Scoping Review

**DOI:** 10.2196/26709

**Published:** 2021-10-20

**Authors:** Dylan P Griswold, Laura Fernandez, A M Rubiano

**Affiliations:** 1 Department of Clinical Neurosciences University of Cambridge Cambridge United Kingdom; 2 NIHR Global Health Research Group on Neurotrauma University of Cambridge Cambridge United Kingdom; 3 Neuroscience Institute INUB-MEDITECH Research Group El Bosque University Bogota Colombia; 4 Neurological Surgery Service Vallesalud Clinic Cali Colombia

**Keywords:** diagnostic criteria, management, neurosurgery, neurotrauma, SAH, scoping review, TBI, trauma

## Abstract

**Background:**

Globally, 69 million people suffer from traumatic brain injury (TBI) each year and TBI is the most common cause of subarachnoid hemorrhage (SAH). Traumatic SAH (TSAH) has been described as an adverse prognostic factor leading to progressive neurological deterioration and an increase in morbidity and mortality, but there are a limited number of studies which evaluate recent trends in the diagnostic and management of SAH in the context of trauma.

**Objective:**

The objective of this scoping review was to understand the extent and type of evidence in relation to the diagnostic criteria and management of TSAH.

**Methods:**

This scoping review will be conducted in accordance with the Joanna Briggs Institute methodology for scoping reviews. A 3-step search strategy (an initial limited search in PubMed and Scopus databases; a main search of EMBASE, Web of Science, EBSCO, MEDLINE; and manual searches of reference lists of included articles) will be utilized. The search will be limited to studies with human participants and published in English, Spanish, and French between 2005 and 2020. This review will consider studies of adolescent and adult patients with SAH secondary to trauma. Study selection will be performed by 2 authors (DG and LF) in a 2-phase process; if any disagreement arises, a third author (AR) will be consulted. Data to be extracted from each study will include population, intervention, comparator and outcome measures, and a summary of findings. Citation screening, full-text review, risk of bias assessment, and extraction of study characteristics and outcomes will be carried out using a web-based software platform that streamlines the production of scoping reviews.

**Results:**

Ethics approval is not required for this systematic review, as there will be no patient involvement. The search for this systematic review commenced in December 2020, and we expect to publish the findings in early 2021. The plan for dissemination is to publish review findings in a peer-reviewed journal and present findings at conferences that engage the most pertinent stakeholders.

**Conclusions:**

This scoping review will serve as an initial step in providing more evidence for health care professionals, economists, and policymakers so that they might devote more resources toward this significant problem affecting both health and economic outcomes worldwide.

**International Registered Report Identifier (IRRID):**

PRR1-10.2196/26709

## Introduction

It is estimated that, globally, 69 million people (95% CI 64-74 million) have a traumatic brain injury (TBI) each year [1]. High-income countries have nearly 3 times more cases than low-and middle-income countries (LMICs) [1]. This is relevant because TBI is the most common cause of subarachnoid hemorrhage (SAH). Thus, traumatic subarachnoid hemorrhage (TSAH) is a common finding in moderate and severe TBI (sTBI), because it occurs in 33%-60% of patients [2,3]. Road traffic accidents, falls, and violence are the main contributing factors to sTBI, and the majority of victims are in the prime of life (aged 15-44) and leading contributors to the country’s gross domestic product (GDP). Thus, a country’s economic security is affected by sTBI, and the country should have a vested interest in reducing its prevalence [4].

Although it is necessary to understand this condition’s pathophysiology more completely, some theories have been described in animal studies that could largely explain the clinical course of TSAH. These theories are principally concerned with the phenomenon of traumatic vasoconstriction, which contributes to secondary ischemic damage and has a variable incidence range of 19%-68%. Marmarou and associates [5] and Thomas and colleagues [6] used a rat model to describe the significant increase of intracranial pressure and mean arterial blood pressure changes that occur as a compensatory mechanism to maintain normal cerebral perfusion pressure [2].

TSAH has been described as an adverse prognostic factor leading to progressive neurological deterioration and increased morbidity and mortality. This is because of its related events of vasospasm, dyselectrolytemia, pituitary dysfunction, hypoxia, intracranial hypertension, and hydrocephalus [3].

Current resources aim to understand the diagnosis and treatment of patients with SAH according to the severity degree of the trauma. The goal is to use this information to evaluate the cost-effectiveness of current management, reduce the length of stay, and redirect the use of already limited resources [7]. Recently published studies have mentioned that patients with SAH secondary to mild TBI (mTBI) have a lower risk of clinical deterioration and that surgical intervention [8], along with the routine implementation of computed tomography scans, mandatory neurosurgery consultations, and high-intensity observations, is not necessary in most cases [7,8].

TSAH is a public health problem of significant proportions because of the global burden of disease and its disproportionate effect on LMICs. While research has made it possible to improve the use of resource-stratified clinical interventions, it is not enough [7]. Economies are dependent on fiscally active adults, and TSAH stunts the growth of GDP in LMICs. The implications, then, lie beyond the scope of medicine and must be taken up by economists and politicians.

The bone structure, clinical outcomes, and pathophysiology of TBI in the pediatric population differ from adults and thus were excluded from the review [9]. Therefore, the objective of this scoping review is to develop a better understanding of TSAH in the adult population. This scoping review will serve as an initial step in providing more evidence for health care professionals, economists, and policymakers so that they might devote more resources toward this significant problem affecting both health and economic outcomes worldwide.

A preliminary search of MEDLINE, the Cochrane Database of Systematic Reviews, and Joanna Briggs Institute (JBI) Evidence Synthesis was conducted, and no current or under way systematic reviews or scoping reviews on the topic were identified.

## Methods

**Figure 1:**
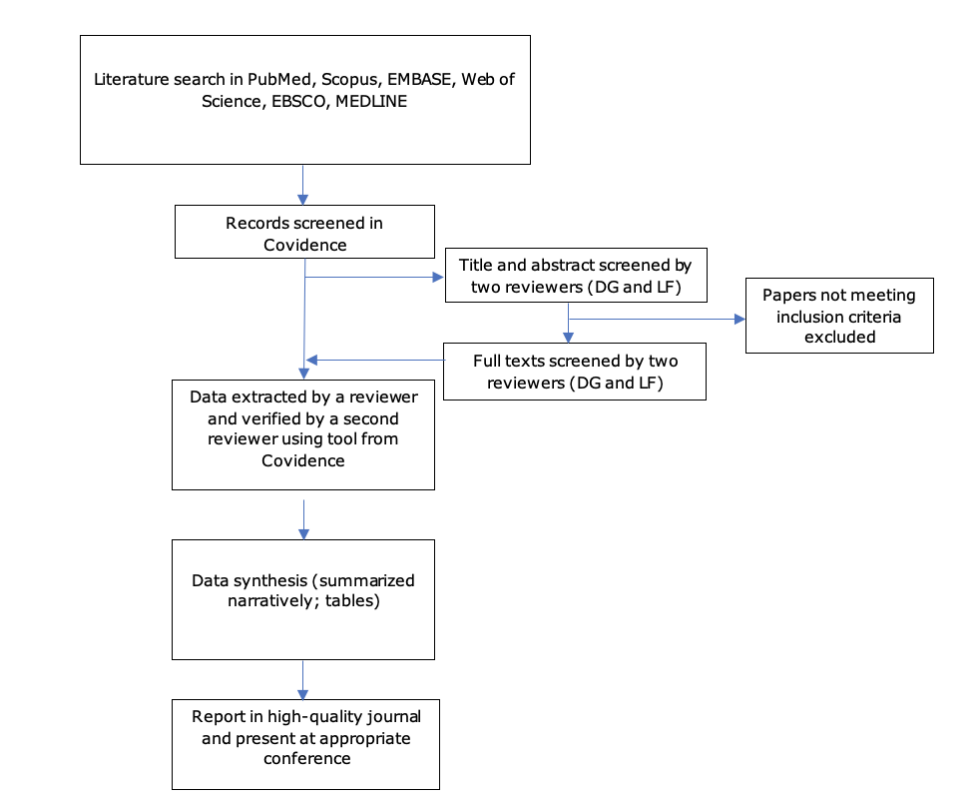
Summary of search strategy process.

The proposed scoping review will be conducted in accordance with the JBI methodology for scoping reviews [10]. The proposed methodology is presented in Figure 1.

### Search Strategy

The search strategy will aim to locate both published and unpublished studies. An initial limited search of MEDLINE and Scopus was undertaken to identify articles on the topic. The text words contained in the titles and abstracts of relevant articles, and the index terms used to describe the articles were used to develop a full search strategy for PubMed and Scopus. A full search strategy for both MEDLINE and SCOPUS is detailed in Multimedia Appendices 1 and 2, respectively. In the second phase of the search, a final search strategy will be adopted for each information source. The reference lists of all selected studies will be screened for additional studies during the third phase of the search.

Studies published in English, Spanish, and French between the years 2005 and 2020 will be included.

### Review Question and Keywords

What is the current evidence on the diagnostic and management protocols of traumatic subarachnoid hemorrhage?

The following keywords will be used: diagnostic criteria; management; neurosurgery; neurotrauma; SAH; scoping review; TBI; trauma

### Inclusion Criteria

#### Participants

Studies of adult and teenage (>15 years old) patients will be included. All studies of pediatric patients (<15 years old) will be excluded.

#### Concept

The concept of interest for the proposed scoping review is studies of subarachnoid hemorrhage secondary to TBI. This will include, but not be limited to, population, intervention, validation status, method of study development, whether the study is consensus or evidence based in addition to the comparator and outcome measures. All studies focused on non-TSAH will be excluded.

#### Context

The review will be limited to studies conducted between 2005 and 2020 in keeping with the objective to evaluate recent trends.

#### Types of Sources

This scoping review will consider both experimental and quasi-experimental study designs including randomized controlled trials, nonrandomized controlled trials, before and after studies, and interrupted time-series studies. Besides, analytical observational studies, including prospective and retrospective cohort studies, case–control studies, and analytical cross-sectional studies will be considered for inclusion. This review will also consider descriptive observational study designs including case series, individual case reports, and descriptive cross-sectional studies for inclusion.

Qualitative studies will also be considered that focus on qualitative data including, but not limited to, designs such as phenomenology, grounded theory, qualitative description, and action research.

In addition, systematic reviews that meet the inclusion criteria will be considered, depending on the research question.

Text and opinion papers will also be considered for inclusion in this scoping review.

### Information Sources

The databases to be searched include PubMed, Scopus, EMBASE, Web of Science, EBSCO, and MEDLINE.

### Study/Source of Evidence Selection

Following the search, all identified citations will be collated and uploaded into EndNoteX9 (Clarivate Analytics). The citations will then be imported into Covidence online software (Veritas Health Innovation) for screening. Two independent researchers (DG and LF) will examine titles and abstracts for inclusion. The full text of selected studies will be retrieved and assessed. Full-text studies that do not meet the inclusion criteria will be excluded, and the reasons for exclusion will be provided in an appendix in the final scoping review. Any disagreements that arise between the researchers during either title and abstract screening or full-text screening will be resolved through discussion, or with a third reviewer (AR). Included studies will undergo a process of data extraction. The results of the search will be reported in full in the final article and presented using the Preferred Reporting Items for Systematic Reviews and Meta-Analyses extension for Scoping Reviews (PRISMA-ScR) checklist.

### Data Extraction

Data will be extracted from the papers included in the review by 2 independent researchers (DG and LF) using the data extraction instrument (Multimedia Appendix 3). The following information will be extracted from the articles: (1) study title; (2) aim; (3) country; (4) methodology; (5) duration; (6) participant characteristics; (7) intervention; (8) outcome measures; (9) summary of findings; and (10) recommendations for future development.

The draft data extraction tool will be modified and revised as necessary during the process of extracting data from each included study. Modifications will be detailed in the full scoping review report. Any disagreements that arise between the reviewers will be resolved through discussion, or with a third reviewer (AR). Authors of papers will be contacted to request missing or additional data, where required.

### Data Analysis and Presentation

The extracted data will be presented in tabular form and as a narrative summary that aligns with the aim of this scoping review. The table will report: (1) distribution of studies by countries of origin/study design; (2) participants/sample size; (3) intervention studied; (4) outcome measure; and (5) summary of findings. This table may be further refined at the review stage. Graphical representations may be used, including bar charts, line charts, pie charts, and diagrams. A narrative summary will accompany the tabulated or charted results and will describe how the results relate to the review’s objectives.

## Results

No ethical approval will be required, as this review is based on already published data and does not involve interaction with human participants. The search for this systematic review commenced in December 2020, and we expect to publish the findings in early 2021. The plan for dissemination, however, is to publish the findings of the review in a peer-reviewed journal and present findings at high-level international conferences that engage the most pertinent stakeholders.

## Discussion

This protocol has been rigorously developed and designed specifically to identify and summarize the available literature regarding the diagnosis and management of TSAH. The results from this scoping review will serve as an initial step to provide greater evidence for health care professionals, economists, and policymakers to encourage them to devote more resources toward this significant problem affecting both health and economic outcomes worldwide. Preliminarily, we have observed that there is a paucity of information available for TSAH associated with sTBI and the evidence on TSAH. Furthermore, the evidence on mTBI greatly outweighs that which is available for TSAH and sTBI.

## Appendix

Multimedia Appendix 1 PubMed search strategy.

Multimedia Appendix 2 Scopus search strategy.

Multimedia Appendix 3 Data extraction instrument.

## Abbreviations

GDP: gross domestic product
JBI: Joanna Briggs Institute
LMIC: low-and middle-income country
mild TBI: mild traumatic brain injury
SAH: subarachnoid hemorrhage
sTBI: severe traumatic brain injury
TBI: traumatic brain injury
TSAH: traumatic subarachnoid hemorrhage

## References

[1] Dewan Michael C, Rattani Abbas, Gupta Saksham, Baticulon Ronnie E, Hung Ya-Ching, Punchak Maria, Agrawal Amit, Adeleye Amos O, Shrime Mark G, Rubiano Andrés M, Rosenfeld Jeffrey V, Park Kee B (2018). Estimating the global incidence of traumatic brain injury. J Neurosurg.

[2] Armin SS, Colohan ART, Zhang JH (2013). Traumatic subarachnoid hemorrhage: our current understanding and its evolution over the past half century. Neurological Research.

[3] Modi N, Agrawal M, Sinha V (2016). Post-traumatic subarachnoid hemorrhage: A review. Neurol India.

[4] Bruns J, Hauser W (2003). The epidemiology of traumatic brain injury: a review. Epilepsia.

[5] Marmarou A, Foda M, van den Brink W, Campbell J, Kita H, Demetriadou K (1994). A new model of diffuse brain injury in rats. Part I: Pathophysiology and biomechanics. J Neurosurg.

[6] Thomas S, Tabibnia F, Schuhmann M, Brinker T, Samii M (2000). ICP and MABP following traumatic subarachnoid hemorrhage in the rat. Acta Neurochir Suppl.

[7] Cooper SW, Bethea KB, Skrobut TJ, Gerardo R, Herzing K, Torres-Reveron J, Ekeh AP (2019). Management of traumatic subarachnoid hemorrhage by the trauma service: is repeat CT scanning and routine neurosurgical consultation necessary?. Trauma Surg Acute Care Open.

[8] Phelan HA, Richter AA, Scott WW, Pruitt JH, Madden CJ, Rickert KL, Wolf SE (2014). Does isolated traumatic subarachnoid hemorrhage merit a lower intensity level of observation than other traumatic brain injury?. J Neurotrauma.

[9] Araki Takashi, Yokota Hiroyuki, Morita Akio (2017). Pediatric Traumatic Brain Injury: Characteristic Features, Diagnosis, and Management. Neurol Med Chir (Tokyo).

[10] Aromataris E, Munn Z, Tufanaru C, Campbell J, Hopp L (2014). JBI Reviewers’ Manual.

