# Supplementary material for: Diagnosis and Management of Traumatic Subarachnoid Hemorrhage: Protocol for a Scoping Review
Source: JMIR Res Protoc. 2021 Oct 20;10(10):e26709. doi: 10.2196/26709 (PMC8567149; doi:10.2196/26709)
Supplement: Multimedia Appendix 1 [file resprot_v10i10e26709_app1.docx]

| **MEDLINE** | **Query** | **Records Retrieved^a^** |
| --- | --- | --- |
| #1 | (("injuries"[MeSH Subheading] OR "injuries"[All Fields] OR "trauma"[All Fields] OR "wounds and injuries"[MeSH Terms] OR ("wounds"[All Fields] AND "injuries"[All Fields]) OR "wounds and injuries"[All Fields] OR "trauma s"[All Fields] OR "traumas"[All Fields]) AND ("subarachnoid haemorrhage"[All Fields] OR "subarachnoid hemorrhage"[MeSH Terms] OR ("subarachnoid"[All Fields] AND "hemorrhage"[All Fields]) OR "subarachnoid hemorrhage"[All Fields]) AND ("diagnosable"[All Fields] OR "diagnosi"[All Fields] OR "diagnosis"[MeSH Terms] OR "diagnosis"[All Fields] OR "diagnose"[All Fields] OR "diagnosed"[All Fields] OR "diagnoses"[All Fields] OR "diagnosing"[All Fields] OR "diagnosis"[MeSH Subheading])) OR (("injuries"[MeSH Subheading] OR "injuries"[All Fields] OR "trauma"[All Fields] OR "wounds and injuries"[MeSH Terms] OR ("wounds"[All Fields] AND "injuries"[All Fields]) OR "wounds and injuries"[All Fields] OR "trauma s"[All Fields] OR "traumas"[All Fields]) AND ("subarachnoid haemorrhage"[All Fields] OR "subarachnoid hemorrhage"[MeSH Terms] OR ("subarachnoid"[All Fields] AND "hemorrhage"[All Fields]) OR "subarachnoid hemorrhage"[All Fields]) AND ("diagnosable"[All Fields] OR "diagnosi"[All Fields] OR "diagnosis"[MeSH Terms] OR "diagnosis"[All Fields] OR "diagnose"[All Fields] OR "diagnosed"[All Fields] OR "diagnoses"[All Fields] OR "diagnosing"[All Fields] OR "diagnosis"[MeSH Subheading])) | 3,430 |
| Filters | **Humans, English, French, Spanish from 2005 - 2020** | 1,678 |

^a^Search conducted on 09 December 2020
