# Supplementary material for: Diagnosis and Management of Traumatic Subarachnoid Hemorrhage: Protocol for a Scoping Review
Source: JMIR Res Protoc. 2021 Oct 20;10(10):e26709. doi: 10.2196/26709 (PMC8567149; doi:10.2196/26709)
Supplement: Multimedia Appendix 2 [file resprot_v10i10e26709_app2.docx]

| **Scopus** | **Query** |
| --- | --- |
| #1 | ((INDEXTERMS(injuries) OR ALL(injuries) OR ALL(trauma) OR INDEXTERMS("wounds and injuries") OR (ALL(wounds) AND ALL(injuries)) OR ALL("wounds and injuries") OR ALL("trauma s") OR ALL(traumas)) AND (ALL("subarachnoid haemorrhage") OR INDEXTERMS("subarachnoid hemorrhage") OR (ALL(subarachnoid) AND ALL(hemorrhage)) OR ALL("subarachnoid hemorrhage")) AND (ALL(diagnosable) OR ALL(diagnosi) OR INDEXTERMS(diagnosis) OR ALL(diagnosis) OR ALL(diagnose) OR ALL(diagnosed) OR ALL(diagnoses) OR ALL(diagnosing) OR INDEXTERMS(diagnosis))) OR ((INDEXTERMS(injuries) OR ALL(injuries) OR ALL(trauma) OR INDEXTERMS("wounds and injuries") OR (ALL(wounds) AND ALL(injuries)) OR ALL("wounds and injuries") OR ALL("trauma s") OR ALL(traumas)) AND (ALL("subarachnoid haemorrhage") OR INDEXTERMS("subarachnoid hemorrhage") OR (ALL(subarachnoid) AND ALL(hemorrhage)) OR ALL("subarachnoid hemorrhage")) AND (ALL(diagnosable) OR ALL(diagnosi) OR INDEXTERMS(diagnosis) OR ALL(diagnosis) OR ALL(diagnose) OR ALL(diagnosed) OR ALL(diagnoses) OR ALL(diagnosing) OR INDEXTERMS(diagnosis))) |
| Filters | **Humans, English, French, Spanish from 2005 to 2020** |
