# Supplementary material for: Diagnosis and Management of Traumatic Subarachnoid Hemorrhage: Protocol for a Scoping Review
Source: JMIR Res Protoc. 2021 Oct 20;10(10):e26709. doi: 10.2196/26709 (PMC8567149; doi:10.2196/26709)
Supplement: Multimedia Appendix 3 [file resprot_v10i10e26709_app3.docx]

| Author | Aim of the Study | Country | Methodology | Period | Sample size | Outcome measures | Summary of results |
| --- | --- | --- | --- | --- | --- | --- | --- |
|  |  |  |  |  |  |  |  |
